# Fish in habitats with higher motorboat disturbance show reduced sensitivity to motorboat noise

**DOI:** 10.1098/rsbl.2018.0441

**Published:** 2018-10-03

**Authors:** Harry R. Harding, Timothy A. C. Gordon, Rachel E. Hsuan, Alex C. E. Mackaness, Andrew N. Radford, Stephen D. Simpson

**Affiliations:** 1 School of Biological Sciences, University of Bristol, 24 Tyndall Avenue, Bristol BS8 1TQ, UK; 2 Marine Scotland Science, 375 Victoria Road, Aberdeen AB11 9DB, UK; 3 Biosciences, University of Exeter, Geoffrey Pope Building, Stocker Road, Exeter EX4 4QD, UK

**Keywords:** anthropogenic noise, cichlid, disturbance history, Lake Malawi, motorboat

## Abstract

Anthropogenic noise can negatively impact many taxa worldwide. It is possible that in noisy, high-disturbance environments, the range and severity of impacts could diminish over time, but the influence of previous disturbance remains untested in natural conditions. This study demonstrates the effects of motorboat noise on the physiology of an endemic cichlid fish in Lake Malawi. Exposure to motorboats (driven 20–100 m from fish) and loudspeaker playback of motorboat noise both elevated the oxygen-consumption rate at a single lower-disturbance site, characterized by low historic and current motorboat activity. Repeating this assay at further lower-disturbance sites revealed a consistent effect of elevated oxygen consumption in response to motorboat disturbance. However, when similar trials were repeated at four higher-disturbance sites, no effect of motorboat exposure was detected. These results demonstrate that disturbance history can affect local population responses to noise. Action regarding noise pollution should consider the past, as well as the present, when planning for the future.

## 1. Introduction

Anthropogenic noise is present in many biomes across the planet, elevating overall acoustic energy and creating noises that are characteristically different from naturally occurring sounds [1,2]. Recent work has demonstrated that noise pollution can have a wide range of physiological and behavioural effects on many taxa (see [3,4] for reviews). Consequently, anthropogenic noise is considered a global pollutant that appears in international legislation, including the European Commission Marine Strategy Framework Directive and the US National Environment Policy Act.

To date, most studies investigating the consequences of anthropogenic noise for animals have used response means to test for overall impacts on a cohort of individuals, while largely ignoring the variation around the mean which may be driven by intrinsic characteristics or extrinsic factors [5]. However, individual responses within a generation can be affected by prior experience; for example, organisms might exhibit altered tolerance through habituation, sensitization or hearing threshold shifts, or emigrate because of past disturbances (see [6–8]). Furthermore, population responses may be altered over multiple generations through evolutionary adaptation. Experimental manipulations have shown that repeated exposure to anthropogenic noise can alter short-term responses in several species [8,9]. However, studies are lacking that explore how natural variation in responses is related to the long-term disturbance history of wild populations (see [7,10]).

Motorboat noise is increasing globally [11] and has a range of detrimental behavioural, physiological and fitness impacts on fishes [4,12] However, non-uniform distributions of boat use across space and time mean that fishes are exposed to varying levels of motorboat activity [1]. Here, we investigate how the impact of motorboat noise on a wild endemic cichlid in Lake Malawi is affected by variation in disturbance history. First, at a single site with low historic and current motorboat counts, we test a physiological response (oxygen consumption) to *in situ* exposures of both real motorboats driven around the testing site (hereafter referred to as ‘motorboat disturbance’) and loudspeaker playback of motorboat noise. The effect of motorboat disturbance on oxygen consumption was then tested at three further lower-disturbance sites. Finally, we used the same assay to test the response of fish to motorboat disturbance at four higher-disturbance sites.

## 2. Material and methods

### (a) Study system and sites

Work was conducted during April–July 2016 at Thumbi West Island, Lake Malawi (14°01′ S, 34°49′ E). Motorboat activity on Lake Malawi shows considerable spatial variability, with total usage likely to increase in the near future as a result of both human population increase [13] and development of fishing and tourism industries [14,15]. Adult males of the endemic cichlid *Cynotilapia zebroides* (previously known as *C. afra* and *Microchromis zebroides*) were chosen as the study organism (see electronic supplementary material, Methods).

Sites were classified for disturbance levels based on analysis of both historic and current motorboat activity (full details in electronic supplementary material, Methods). Trip logs from the area's two local dive operators, which are representative of local nearshore motorboat traffic, were used to identify four lower-disturbance and four higher-disturbance sites. Boat counts of all vessel traffic confirmed that historic patterns matched current motorboating activity; at the time of the study, there were 10 times more boat-passes at higher-disturbance sites than lower-disturbance sites.

### (b) Acoustic stimuli

For playbacks, 10 independent 5-min underwater recordings of daytime ambient conditions (five different times of day) and motorboat noise (five different boats) were taken at the initial lower-disturbance site (see electronic supplementary material, Methods for details on playback-track creation and sound-level adjustment, which followed the methods in [16]). For the actual motorboat disturbance exposures, eight different boats were used across the eight sites (1–5 boats per site). Representative recordings of ambient conditions and motorboat noise were taken at each experimental site at the location of the fish during the trials. All recordings were analysed in both sound-pressure and particle-motion domains (electronic supplementary material, figure S1 and methods for full details).

### (c) Identifying impacts of motorboat noise at a single lower-disturbance site

Oxygen-consumption rate is an emerging physiological tool for understanding likely impacts of anthropogenic pollutants on ecosystems [17]. The effect of motorboat noise on the oxygen-consumption rate of *C. zebroides* was tested *in situ* using an independent-measures experimental design. Oxygen-consumption rates were compared between fish exposed to either ambient conditions, motorboat disturbance or their playback equivalents. The complementary use of real motorboats and loudspeaker playback allowed both acoustic validity and isolation of motorboat noise as a stressor independent of visual cues and wake effects.

Fish were placed into an open-ended container for an acclimation period of 5 min before the container was sealed underwater in the lake and the sound treatment started; trials lasted for 30 min, with four fish run in parallel during each trial (full details in electronic supplementary material, Methods). Sealed containers were opaque, eliminating visual cues associated with exposure to motorboat disturbance. Water temperature and dissolved oxygen content in containers was measured at the start and end of the trial (Dissolved Oxygen and Temperature Meter HI 9164, Hanna Instruments Inc., Woonsocket, USA), and fish length and mass were recorded immediately after trials. Oxygen content and mass data were used to calculate oxygen-consumption rates of fish over the trial period (mg O_2_ g^–1^ h^–1^) To assess the impact of motorboat noise, sound treatment (ambient sound or motorboat noise) and sound source (real sound or loudspeaker playback), and their interaction, were included as predictor variables in a two-way ANOVA.

### (d) Testing for effects of motorboat-disturbance history through multi-site comparisons

**Figure 1.**
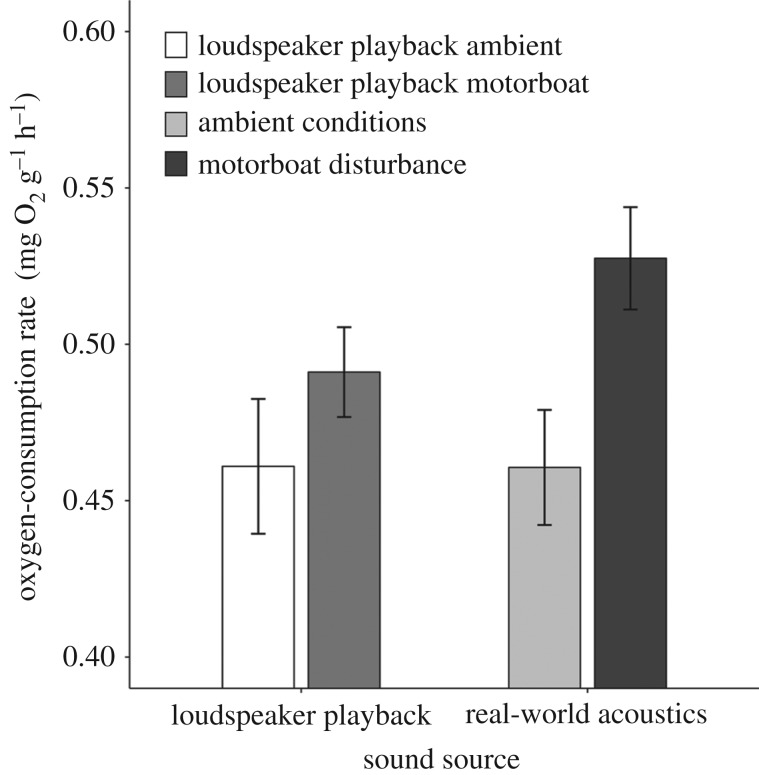
Mean ± s.e. oxygen-consumption rate in *C. zebroides* exposed to playback of ambient sound (*n* = 19), playback of motorboat noise (*n* = 20), ambient conditions (*n* = 18) or motorboats (*n* = 19). Sound treatment (ambient sound or motorboat noise) had a significant effect, but the sound source (real sound or loudspeaker playback) did not.

Having established qualitatively equivalent responses to motorboat disturbance and motorboat-noise playback (figure 1), motorboat disturbance was used exclusively for the multi-site comparisons to achieve acoustic validity. Assays were conducted at three additional lower-disturbance sites to investigate whether the oxygen-consumption response detected at the initial lower-disturbance site was consistently found. Assays were then conducted at four higher-disturbance sites to test whether the same response was apparent. Linear mixed models (LMMs) were used to control for the testing of multiple fish from the same sites.

## 3. Results

At the initial lower-disturbance site, there was a significant effect of sound treatment: fish exposed to motorboat noise exhibited higher oxygen-consumption rates than those exposed to ambient sound (two-way ANOVA: *F*_1,72_ = 8.42, *p* = 0.005; figure 1). However, there was no significant effect of sound source (real sound versus loudspeaker playback) (*F*_1,72_ = 1.17, *p* = 0.28), and no significant interaction between sound treatment and sound source (*F*_1,72_ = 0.80, *p* = 0.37).

**Figure 2.**
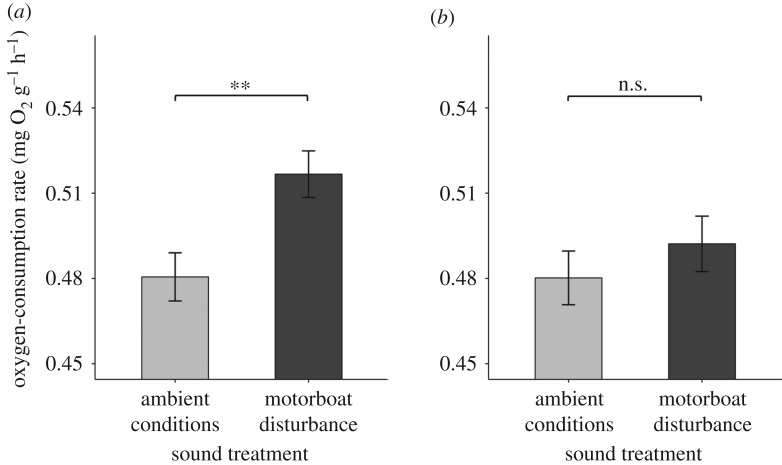
Mean ± s.e. oxygen-consumption rate in *C. zebroides* exposed to ambient conditions or motorboat disturbance in areas with (*a*) lower (ambient: *n* = 72; motorboats: *n* = 69) or (*b*) higher (ambient: *n* = 71; motorboats: *n* = 70) current and historic levels of motorboat activity. **p ≤ 0.01; n.s. denotes no significant difference.

The significant increase in oxygen-consumption rate in response to motorboat disturbance found at the initial lower-disturbance site was replicated when considering all four lower-disturbance sites (LMM: *χ*^2^ = 9.239, *df* = 1, *p* = 0.002, intercept (ambient conditions) ± s.e. = 0.481 ± 0.008, effect size ± s.e. = 0.036 ± 0.012; figure 2*a*). However, there was no significant effect of motorboat disturbance on the oxygen-consumption rate at the four higher-disturbance sites (*χ*^2^ = 0.786, *df* = 1, *p* = 0.375, intercept (ambient conditions) ± s.e. = 0.480 ± 0.010, effect size ± s.e. = 0.012 ± 0.014; figure 2*b*). The variance associated with the random ‘Site ID’ term was less than 0.001 in both cases (variance ± s.d.: lower-disturbance less than 0.001 ± less than 0.001; higher-disturbance less than 0.001 ± 0.008).

## 4. Discussion

This study used a rapid-assessment physiological assay to demonstrate that responses to motorboat noise in wild endemic cichlids in Lake Malawi were lower in areas with higher levels of motorboat disturbance. This represents novel *in situ* evidence to add to a small but increasing body of work examining intraspecific variation in organismal responses to noise. Such variation can occur due to both intrinsic characteristics (e.g. physiological body condition) and external factors (e.g. prior exposure) [8,10,18,19].

The ecological equivalence of all sites in this study (matched by depth, distance to shore, benthic substrate and water temperature) suggests that the observed difference in response to noise was the result of differences in disturbance history. In contrast to previous laboratory-based extended exposures of fishes to noise [8], the lower response of fish at natural higher-disturbance sites could represent either acclimation within a generation (e.g. increased tolerance through shifts in hearing sensitivity thresholds, or a declining response from learning that the stimulus does not have any detrimental consequences [6,7]) and/or adaptation through selection over multiple generations (e.g. [20]). Indeed, tolerance may itself be a phenotypic trait subject to plasticity; for example, organisms with a higher tolerance of noise may have a selective advantage in high-disturbance areas through increased opportunities for foraging and mating [10,20]. Future work could use extended field-based manipulations of motorboat exposure within a generation to isolate disturbance history from any unmeasured and potentially confounding variables. This would facilitate further understanding of both the mechanistic drivers and the timescale over which such changes in tolerance develop [7].

Our study provides evidence from wild fishes that physiological responses to motorboats can be affected by existing variation in disturbance history, with the equivalent result shown in response to real motorboat and playbacks suggesting a strong influence of noise. Such intraspecific variation has implications for understanding and mitigating effects of noise on wildlife; for example, ecosystem impact assessments carried out in historically disturbed areas may represent underestimates of the threats posed to wider populations by novel sources of noise pollution. As such, we advocate further work that moves beyond asking simply whether there is an impact of noise pollution, but also considers the causes of variation in the response.

## Supplementary Material

Supplementary Methods

## Data accessibility

Data available from the Dryad Digital Repository: http://dx.doi.org/10.5061/dryad.8ss7096 [21].
