# Supplementary material for: Fish in habitats with higher motorboat disturbance show reduced sensitivity to motorboat noise
Source: Biol Lett. 2018 Oct 3;14(10):20180441. doi: 10.1098/rsbl.2018.0441 (PMC6227867; doi:10.1098/rsbl.2018.0441)
Supplement: Supplementary Methods [file rsbl20180441supp1.docx]

**Fish in habitats with higher motorboat disturbance show reduced sensitivity to motorboat noise**

Harry R. Harding, Timothy A. C. Gordon, Rachel E. Hsuan, Alex C. E. Mackaness, Andrew N. Radford, Stephen D. Simpson

**Supplementary Methods**

*Study system and sites*

The endemic cichlid *Cynotilapia zebroides* was chosen as the study organism based on: its abundance at the study site; its distinctive colouration allowing accurate identification despite the high species diversity and multiple intra-population colour polymorphisms present in the Lake Malaŵi species complex [S1, S2]; and previous evidence that acoustic cues play important roles in the ecology of similar cichlid species [S3, S4]. *C. zebroides* at Thumbi West Island represent a rapidly evolving population that are hybridising with the local *Metriaclima zebra* population [S2]; this is the case for all studied fish at all experimental sites.

Trip logs from both of the only two local dive operators (*Cape Maclear SCUBA* and *SCUBA Shack*), which represented all formally logged motorboat use in the area, were used to identify four lower-disturbance sites (no recorded visits in the last 3 years) and four higher-disturbance sites (visited in more than 8 of the last 12 months; mean ± SE, 51 ± 13 logged dives in 33 months) around the island. Combined visits to the four higher-disturbance sites comprised over 60% of total logged motorboating activity, and none of the four lower-disturbance sites ever appeared in either log. These logs provided a quantifiable and representative assessment of general patterns of local near-shore motorboat traffic by a much larger fleet of similar, unlogged vessels, since other motorboat users follow the leads of these dive operators when taking tourists to see fishes.

Counts of current activity includes all motorboat traffic in the area passing within 50 m of the selected sites (distance estimated visually; at least two counts per site with a total time of at least 4 h). Higher-disturbance sites (mean ± SE: 3.58 ± 0.82 boats/hr) experienced 10 times more passes per hour on average than lower-disturbance sites (0.37 ± 0.19 boats/hr), and the passes were over twice as close to shore (mean ± SE, higher-disturbance sites: 19 ± 1.4 m; lower-disturbance sites: 45 ± 1.6 m). Boat counts were made between 10:30 and 17:00, with no difference in times between higher- and lower-disturbance sites. All selected sites were acoustically independent, shallow (2–5 m) coastal areas with a rocky benthic substrate, separated by a body of water with a minimum distance of 300 m and/or a prominent headland (see Fig. S2 for the locations of the eight sites around Thumbi Island West).

*Acoustic stimuli*

A single representative 5-min recording of the ambient conditions and motorboat noise used in the experiments was taken at each site. Recordings for playback experiments were taken in 2 m depth, 10 m from the shore. Ambient conditions had no boat traffic within 500 m. Motorboats were all of the type most commonly used in the area (7-m-long wooden hulls with 8–15 horsepower rear-mounted outboard engines), driven at various speeds 20–100 m from the recording equipment, as per [S5, S6].

Acoustic pressure was measured with a calibrated omnidirectional hydrophone (HiTech HTI-96-MIN with inbuilt preamplifier, manufacturer-calibrated sensitivity -164.3 dB re 1V/μPa; frequency range 0.002–30 kHz; calibrated by manufacturers; High Tech Inc., Gulfport MS) connected to a digital recorder (PCM-M10, 48 kHz sampling rate, Sony Corporation, Tokyo, Japan). Particle motion was measured with a calibrated accelerometer (M20L; sensitivity following a curve over the frequency range 0–2 kHz; calibrated by manufacturers; Geospectrum Technologies, Dartmouth, Canada) connected to a digital 4-track recorder (Boss BR-800, 44.1 kHz sampling rate, Roland Corporation, Los Angeles, CA) and expressed as acceleration. Recording levels were calibrated using 1 kHz pure sine wave signals from a function generator with a measured voltage recorded in line on an oscilloscope. Sound-recording equipment was positioned 1 m above the lake bed attached to an inflatable raft or a submerged stand to avoid unwanted noise from waves on the hull of a rigid boat. The accelerometer was suspended by rope to reduce mechanical noise affecting the particle-motion recordings.

All tracks for playback experiments were created using Audacity 2.1.2 (www.audacityteam.org), and adjusted to ensure that the root-mean-squared average amplitude levels (used as a measure of track volume and analysed using SASLabPro v5.2.07; Avisoft Bioacoustics) received during trials were equivalent to those received in original recordings. The sound system for playbacks consisted of a loudspeaker (University Sound UW-30; maximal output 156 dB re 1 μPa at 1 m, frequency response 0.1–10 kHz; Lubell Labs, Columbus, OH) positioned 3 m from the trial, an amplifier (M033N, 18 W, frequency response 0.04–20 kHz; Kemo Electronic GmbH, Germany), an MP3 player (BUSH, New Mexico, USA), and a battery (12v 12Ah sealed lead-acid; CSB Co. Ltd, Vietnam). Representative recordings of ambient sounds and motorboats were made at all sites for the multi-site comparison in 1.5–3 m depth, 6–13 m from shore.

All recordings were analysed in both sound-pressure and particle-motion domains using MATLAB 2013a (MathWorks Inc., PAMGuide & paPAM analysis packages [S13–S14]). Power spectral densities were determined across the likely hearing range of cichlids; this was estimated as being the frequency range 0–2 kHz, based on previous studies involving electrophysiological measurements of hearing ability [S7–S9] and analysis of the frequency of courtship-associated cichlid vocalisations [S8, S10, S11]. Playback using loudspeakers alters the characteristics of the original recordings, but analyses of spectral content and sound levels showed that many of the characteristics of the original recordings were retained in playback, and that these characteristics differed between playbacks of ambient and motorboat noise (Fig. S1).

*Identifying impacts of motorboat noise at a single lower-disturbance site*

During all experimental trials, fish were randomly allocated to different sound treatments to avoid selection bias, and the order of tracks or motorboats used was defined randomly within counterbalanced blocks. Treatment order was always alternated to avoid confounding effects of time of day or fish holding-time, and different motorboats and tracks were used in approximately equal numbers of trials.

At the start of each day, male *C. zebroides* were captured at the first lower-disturbance site by snorkelers using a 10 x 1 m barrier net in 2–5 m depth, within a 200 m stretch of coastline. Captured individuals were held during transport and for storage before testing in an opaque barrel containing 20 L of regularly flushed lake water. The testing location was within 500 m of capture, transport was by paddling, and the holding barrel was kept on the shore to ensure acoustic isolation from the experimental trials. Fish were released unharmed to their natal sites at the end of each day, and capture was always over 50 m away from capture sites on previous days; due to the high site-fidelity of territorial Lake Malaŵi cichlids [S12], this avoided the chance of re-testing fish on consecutive days.

To measure oxygen-consumption rate, individual fish (mean ± SE, length: 8.51 ± 0.04 cm; mass: 9.24 ± 0.15 g) were placed in a section of open PVC piping (9.2 x 3.8 cm) with mesh netting over each end to restrict movement, inside a sealed polyethylene terephthalate (PET) container (13.7 x 6.2 cm; 412 ml volume) suspended from a submerged stand 1.5–3 m below the surface of the lake. Acoustic transparency was predicted to be high due to similar acoustic impedance values for water (1.5 MRayls) and PET (1.76 MRayls). Following a 5-min acclimation period, fish were sealed in containers for a 30-min sound-exposure period. Containers were then brought to the surface and fish were removed. All capture, handling and measurement methods were identical for noise-exposed and control fish, facilitating valid comparisons of relative differences in oxygen-consumption rate. There was no significant difference in either length (one-way ANOVA: *F*_3,70_ = 0.38, *p* = 0.77) or mass (Kruskal-Wallis test: *H*_3_ = 0.56, *p* = 0.91) of fish allocated to different sound treatments.

*Testing for effects of motorboat-disturbance history through multi-site comparisons*

Experimental protocols followed those for the initial lower-disturbance site. All experimental sites consisted of a rocky benthic substrate and were characteristically similar in depth (2–6 m), distance to shore (6–13 m) and mean water temperature (23.8–25.3°C). There was no significant difference in trial temperature between treatments in either the higher-disturbance (two-sample t-test: *t* = -0.42, *n_amb_* = 72, *n_boat_* = 69, *p* = 0.67) or lower-disturbance (*n_amb_* = 71, *n_boat_* = 70, *t* = -0.74, *p* = 0.46) sites. There was no significant difference in either mass (Mann-Whitney U-tests, higher-disturbance: *W* = 2401, *n_amb_* = 72, *n_boat_* = 69, *p* = 0.73; lower-disturbance *W* = 2663, *n_amb_* = 71, *n_boat_* = 70, *p* = 0.46) or length (two-sample t-tests, higher-disturbance: t = -0.33, *n_amb_* = 68, *n_boat_* = 64, *p* = 0.74; lower-disturbance: t = 0.76, *n_amb_* = 70, *n_boat_* = 65, *p* = 0.45) of fish allocated to different sound treatments at the lower- and higher-disturbance sites.

Linear mixed models (LMMs) were used to analyse datasets from lower-disturbance and higher-disturbance sites. In both LMMs, sound treatment (ambient or motorboat) was used as a fixed term, site ID (1–4) was included as a random term, and significant effects of sound treatment were confirmed by comparisons with a null model. Visual examination of residual plots never revealed any obvious deviations from homoscedasticity or normality.

**
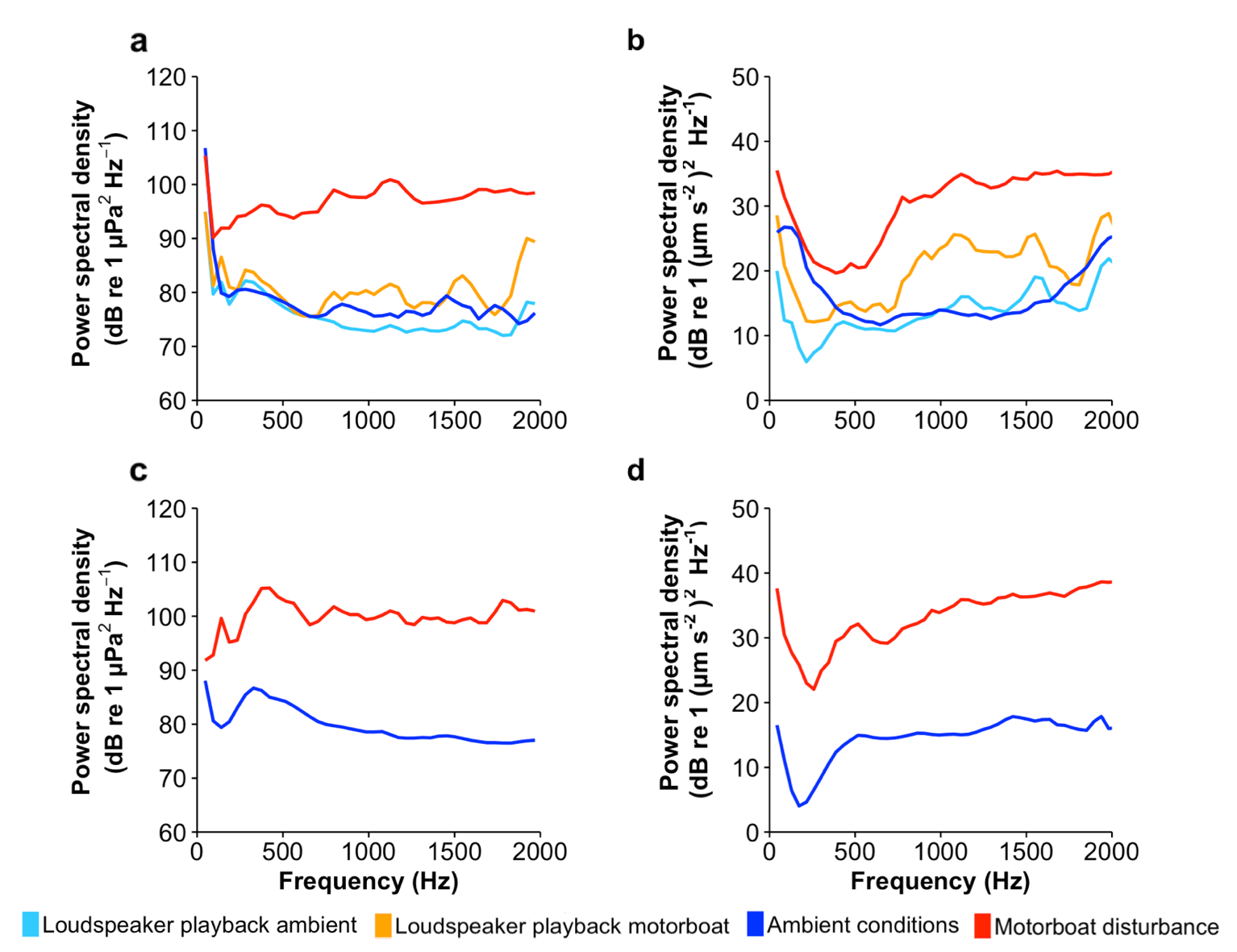
**

**Supplementary Figure S1:** Acoustic analyses of experimental conditions. (a & b) Mean spectral content of combined illustrative recordings of original ambient conditions and motorboat noise, and playback of those recordings in both (a) pressure and (b) particle-motion domains, recorded at the initial single lower-disturbance test site. Root-mean-squared sound-pressure levels (0–2 kHz) and range are: 115.1, 113.3–116.4 (Ambient playback); 117.9, 112.4–121.2 (Motorboat playback); 125.1, 115.4–128.0 (Ambient); and 132.2, 127.5–134.9 (Motorboat disturbance). Particle-acceleration levels (0–2kHz) and range (given in dB re (1µm/s^2^)) are 48.7, 44.6–52.9 (Ambient playback); 55.9, 43.8–62.4 (Motorboat playback); 53.6, 46.7–55.6 (Ambient); and 67.1, 64.1–69.0 (Motorboat disturbance). (c & d) Mean spectral content of combined illustrative recordings of original ambient conditions and motorboat noise taken at each subsequent site, in both (c) pressure and (d) particle-motion domains. Fast Fourier Transform (FFT) analysis of 0–2 kHz, spectrum level units averaged from 30 s recordings, Hamming evaluation window, FFT size = 1024. Root-mean-squared sound-pressure levels (0–2 kHz) and range (given in dB re 1 µPa) are: 115.5, 109.8–120.6 (Ambient); and 135.0, 120.8–142.4 (Motorboat disturbance). Particle-acceleration levels (0–2kHz) and range (given in dB re (1µm/s^2^)) are: 49.1, 46.6–52.2 (Ambient); and 69.3, 62.9–76.8 (Motorboat disturbance).


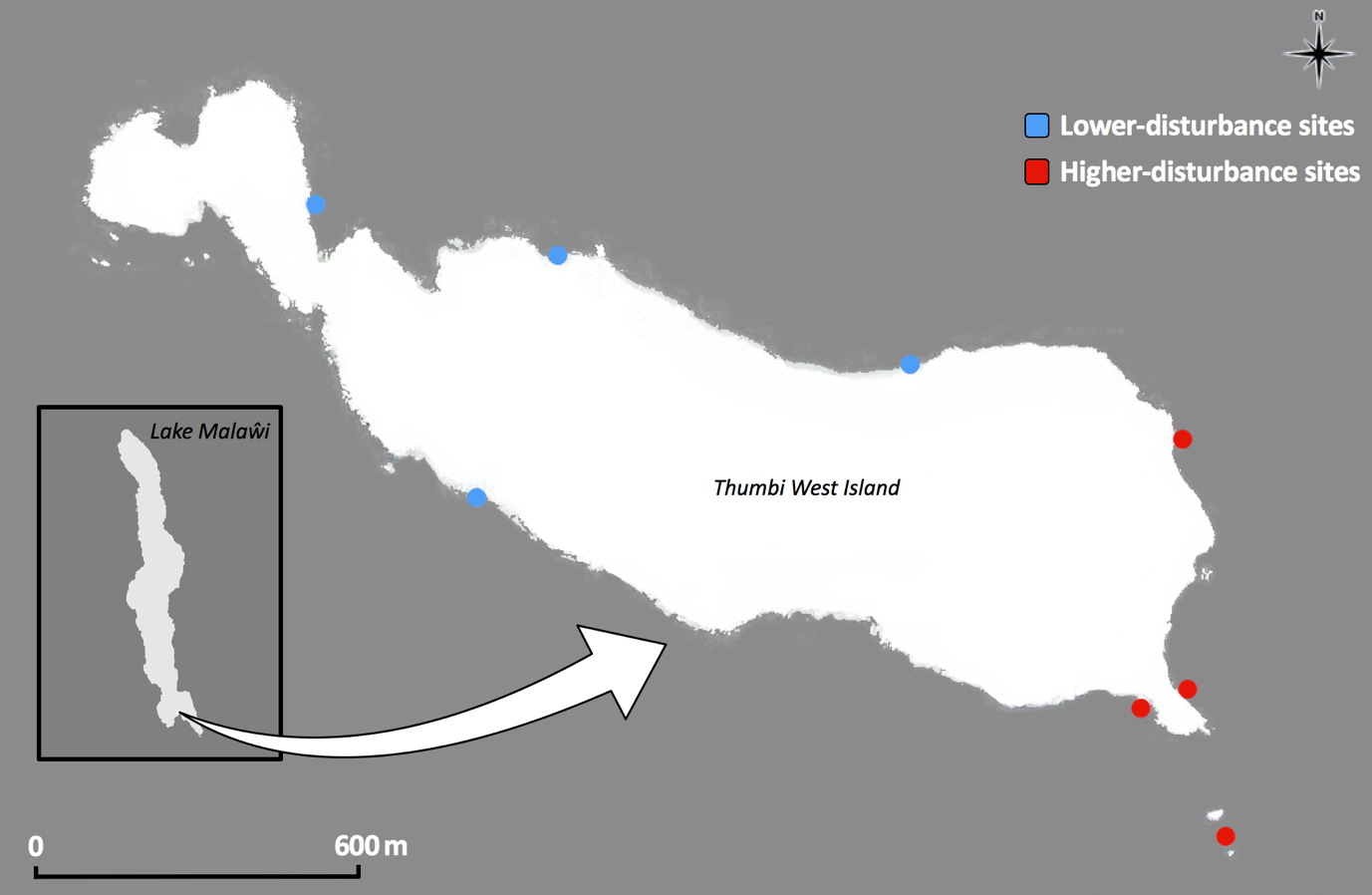


**Supplementary Figure S2:** Image showing the location of the eight sites around *Thumbi West Island* (14° 1’ 14” S, 34° 49’ 9” E). All experimental sites were matched for benthic substrate (rocky bottom), depth (2–6 m), distance to shore (6–13 m) and mean water temperature (23.8–25.3°C).

**References**

S1. Turner GF, Seehausen O, Knight ME, Allender CJ, Robinson RL. 2001 How many species of cichlid fishes are there in African lakes? *Mol. Ecol*. **10**, 793–806. (doi:10.1046/j.1365-294x.2001.01200.x)

S2. Streelman JT, Gmyrek SL, Kidd MR, Kidd C, Robinson RL, Hert E, Ambali AJ, Kocher TD. 2004 Hybridization and contemporary evolution in an introduced cichlid fish from Lake Malaŵi National Park. *Mol. Ecol*. **13**, 2471–2479. (doi:10.1111/j.1365-294X.2004.02240.x)

S3. Bertucci F, Beauchaud M, Attia J., Mathevon N. 2010 Sounds modulate males’ aggressiveness in a cichlid fish. *Ethology* **116**, 1179–1188. (doi:10.1111/j.1439-0310.2010.01841.x)

S4. Bertucci F, Attia J, Beauchaud M, Mathevon N. 2012 Sounds produced by the cichlid fish *Metriaclima zebra* allow reliable estimation of size and provide information on individual identity. *J. Fish Biol*. **80**, 752–766. (doi:10.1111/j.1095-8649.2012.03222.x)

S5. Simpson SD, Radford AN, Nedelec SL, Ferrari MCO, Chivers DP, McCormick MI, Meekan MG. 2016 Anthropogenic noise increases fish mortality by predation. *Nat. Commun*. **7**, 10544. (doi:10.1038/ncomms10544)

S6. Holles SH, Simpson SD, Radford AN, Berten L, Lecchini D. 2013 Boat noise disrupts orientation behaviour in a coral reef fish. *Mar. Ecol. Prog. Ser*. **485**, 295–300. (doi:10.3354/meps10346)

S7. Kenyon TN, Ladich F, Yan HY. 1998 A comparative study of hearing ability in cichlids: the auditory brainstem response approach. *J. Comp. Physiol. A*. **182**, 307–318. (doi:10.1007/s003590050181)

S8. Maruska KP, Ung US, Fernald RD. 2012 The African cichlid fish *Astatotilapia burtoni* uses acoustic communication for reproduction: sound production, hearing, and behavioral significance. *PLoS One* **7**, 1–13. (doi:10.1371/journal.pone.0037612)

S9. Ladich F, Schulz-Mirbach T. 2013 Hearing in cichlid fishes under noise conditions. *PLoS One* **8**, e57588. (doi:10.1371/journal.pone.0057588)

S10. Amorim MCP, Knight ME, Stratoudakis Y, Turner GF. 2004 Differences in sounds made by courting males of three closely related Lake Malawi cichlid species. *J. Fish Biol*. **65**, 1358–1371. (doi:10.1111/j.1095-8649.2004.00535.x)

S11. Amorim MCP, Simões JM, Fonseca PJ, Turner GF. 2008 Species differences in courtship acoustic signals among five Lake Malaŵi cichlid species (*Pseudotropheus spp.*). *J. Fish Biol*. **72**, 1355–1368. (doi:10.1111/j.1095-8649.2008.01802.x)

S12. Hert E. 1992 Homing and home-site fidelity in rock-dwelling cichlids (Pisces: Teleostei) of Lake Malaŵi, Africa. *Environ. Biol. Fishes* **33**, 229–237. (doi:10.1007/BF00005866)

S13. Merchant ND, Fristrup KM, Johnson MP, Tyack PL, Witt MJ, Blondel P, Parks SE. 2015 Measuring acoustic habitats. *Methods Ecol. Evol.* **6**, 257–265. (doi:10.1111/2041-210X.12330)

S14. Nedelec SL, Campbell J, Radford AN, Simpson SD, Merchant ND, Fisher D. 2016 Particle motion: the missing link in underwater acoustic ecology. *Methods Ecol. Evol.* **7**, 836–842. (doi:10.1111/2041-210X.12544)
